# Minimally Invasive Cardiac Surgery: Transapical Aortic Valve Replacement

**DOI:** 10.1155/2012/145381

**Published:** 2012-10-16

**Authors:** Ming Li, Dumitru Mazilu, Keith A. Horvath

**Affiliations:** Cardiothoracic Surgery Research Program, National Heart, Lung, and Blood Institute, National Institutes of Health, 9000 Rockville Pike, Bldg 10, B1D47, Bethesda, MD 20892, USA

## Abstract

Minimally invasive cardiac surgery is less traumatic and therefore leads to quicker recovery. With the assistance of engineering technologies on devices, imaging, and robotics, in conjunction with surgical technique, minimally invasive cardiac surgery will improve clinical outcomes and expand the cohort of patients that can be treated. We used transapical aortic valve implantation as an example to demonstrate that minimally invasive cardiac surgery can be implemented with the integration of surgical techniques and engineering technologies. Feasibility studies and long-term evaluation results prove that transapical aortic valve implantation under MRI guidance is feasible and practical. We are investigating an MRI compatible robotic surgical system to further assist the surgeon to precisely deliver aortic valve prostheses via a transapical approach. Ex vivo experimentation results indicate that a robotic system can also be employed in in vivo models.

## 1. Introduction

Traditional cardiac surgery requires a sternotomy, cardiopulmonary bypass, and cardiac arrest to provide a still and bloodless heart and its vessels for operation. While necessary, these interventions are invasive and traumatic. The morbidity of cardiac surgery can be quite a burden to the patients [1–3].

In order to reduce the risks associated with open-heart surgery, minimally invasive approaches have been investigated [4–7]. Cardiac surgeons operate through small incisions in the chest, eliminating the need for a sternotomy, stopping the heart, or requiring a heart-lung machine to be used. Decreased trauma to tissue and muscle with smaller incisions typically results in less pain. Avoiding the bypass machine reduces the risks for neurological complications and stroke. In general, minimally invasive cardiac surgery, in comparison to traditional procedures, offers many benefits including reduction of the chance for postsurgical complications and leads to shorter hospital stay with a faster return to normal activities.

Aortic valve replacement is such a cardiac procedure that can be performed with minimally invasive techniques. In the last decade, transcatheter aortic valve replacement (TAVR) has been studied for treating the patients of high surgical risk. The bioprosthetic valves are delivered through catheters transfemorally [8–13] or transapically [14–18] and are implanted within the diseased aortic valve. In current clinical practice, the transfemoral approach is the first choice, while the transapical method is only chosen for patients who have poor vascular access [19]. However, the transapical aortic valve approach may be more applicable to a wider range of patients because of the lack of physical anatomic limitations. Antegrade access avoids possible complication with retrograde access, which is caused by inability to cross a stenotic valve. Larger sheath diameters used in the transapical access lead to less need for crimping of the valves, which may be translated into better prosthesis longevity [20, 21]. Early, midterm, clinical, and echocardiographic outcomes indicate that both approaches are comparable [22], despite a significantly higher risk profile in the cohort treated with the transapical approach [23].

Typically, the imaging employed for TAVR is primarily high-resolution fluoroscopy and adjunctive 2-dimensional M-mode transesophageal echocardiography. The problems with fluoroscopy guidance include device embolization, coronary obstruction, low or high placement, misalignment, landmark loss (after ballooning the valve the calcium pattern used by fluoroscopy to identify the leaflets/annulus is changed), perivalvular leaks, need for rapid ventricular pacing, radiation exposure, and intravenous contrast toxicities. All of these are imaging related and may be improved with better imaging; hence our desire is to pursue magnetic resonance imaging guidance.

MRI provides excellent visualization particularly in its ability to provide high-resolution images of blood-filled structures without additional risk of radiation or contrast reaction. Vascular as well as soft tissue visualization can easily be performed simultaneously. MRI also provides the ability to assess ventricular and valvular function and myocardial perfusion. New generations of open, wide, and short bore MR scanners and real-time sequences made MRI not only cardiovascular diagnostics but also minimally invasive cardiac surgery possible.

The confined physical space of the MRI scanner, even with a wider and shorter bore, can be a challenging environment in which valve replacement is performed. During the procedure, the surgeon must manipulate the different components of the delivery device and other tools through the delivery device while visualizing the in-room MRI display simultaneously. In order to deliver the prosthesis properly, a coordinated effort between the surgeon and the team is critical in the noisy MRI environment while contending with respiratory and cardiac motion during a beating heart procedure. The use of a robotic assistance can potentially alleviate the need of this level of coordination and provide dexterous manipulation of the interventional tools inside the MRI scanner. 

Our group has focused on magnetic resonance imaging- (MRI-) guided transapical aortic valve replacement [24–27]. In this paper, we report our work on this beating-heart procedure: surgical techniques, medical imaging, medical devices, feasibility of the procedure, and long-term results. We also report on our work with robotic assistance for this procedure.

## 2. Material and Methods

### 2.1. MR Imaging System

Magnetom Espree (Siemens Medical Solutions, Munich, Germany) is used for the intervention. This 1.5-T magnet design, with short (120 cm) and wide (70 cm) bore, gives a clearance of up to 30 cm above the chest of the supine patient and makes surgical access to the patient within the magnet feasible. In addition to providing standard MR sequences, a fully interactive, rtMRI system connected to the scanner provides a real-time interactive imaging sequencing. This system comprises an interactive user interface, an operating room large-screen display, gated pulse sequences, and image reconstruction software. Multiple oblique slices can be obtained in rapid succession and can be simultaneously displayed in a 3D rendering to provide optimal 3D anatomic information. Image contrast, image plane orientations, acquisition speed, 3D rendering, and device tracking can be readily adjusted as needed during scanning [28]. 

### 2.2. Stents and Devices

**Figure 1:**
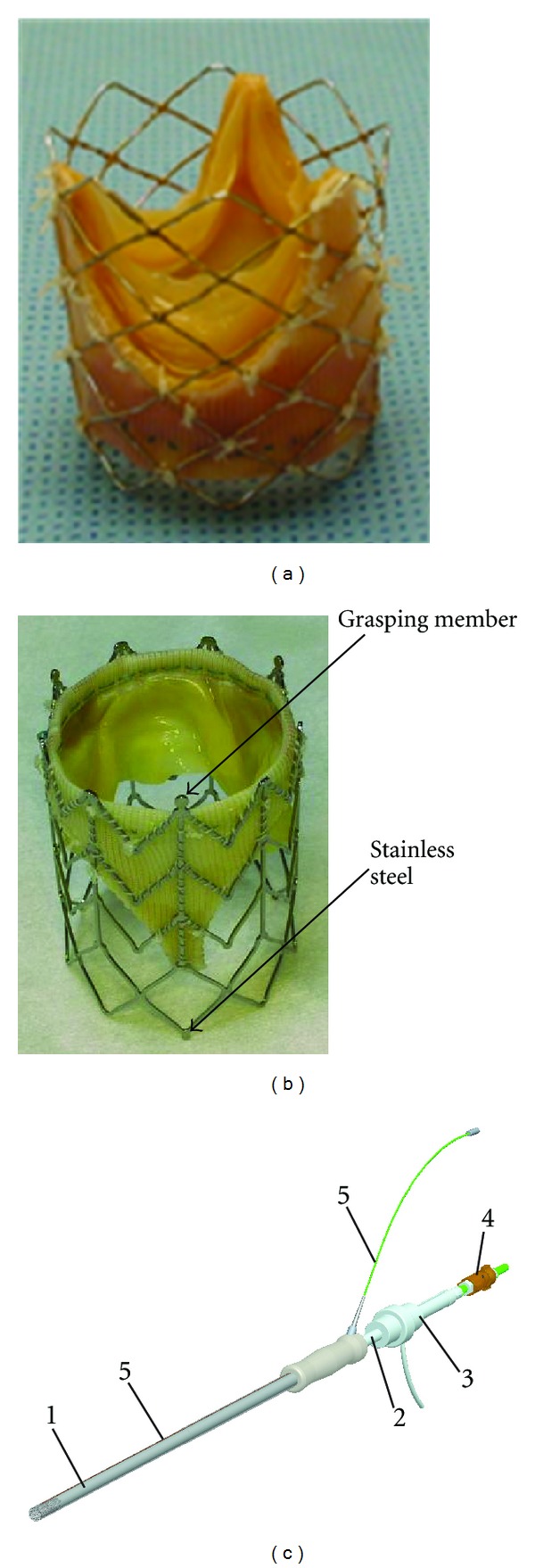
Devices: (a) balloon-expandable prosthesis. (b) Self-expanding prosthesis, a Medtronic Freestyle valve sewn inside the Nitinol self-expanding stent. A small stainless steel welded on the small round extension of distal end of the stent serves as a passive marker indicating the orientation of the prosthesis. The grasping member of the proximal end is used for retrieve and reposition of the stent. (c) A CAD drawing of the delivery device with loop coil and antenna. Sheath with handle (1) protects and retains the prosthesis compressed until the deployment. Inner rod with handle (2) pushes the prosthesis for deployment. Inner channel of the inner rod provides access for the loop snare wire (4). End spacer with dimension protection (3) protects the end valve and ensures the exact dimension for the loop snare wire. The loop coil antenna (5) is fixed into the groove cut on exterior tube of valve delivery system. The delivery device is made of nylon and delrin.

A new self-expanding stent was designed to accommodate conventional stentless aortic bioprostheses (Toronto SPV, St. Jude Medical, Minneapolis, MN, or Freestyle, Medtronic Inc., Minneapolis, MN) [29] (Figure 1). The stent is made of a biocompatible nickel-titanium alloy (nitinol), which assumes a “preprogrammed” final configuration upon release from the delivery system and exposure to body temperature. The stent has nine rods, three of which are aligned with the valve commissures, and a chevron repeating pattern along the length of the cylinder with flared ends. The fixed length of the stent at both crimped and expanded status prevents stress on the bioprosthetic valve especially at suturing area. The chevron geometry also prevents migration of stent at systole (high blood pressure) because of the self-anchoring properties of the chevron spikes. The structure of the stent also makes it easily retractable into a delivery device. Adjustment of the position during valve placement is therefore possible.

We also implanted balloon-expandable bioprostheses. A stentless bioprosthesis (Toronto SPV or Freestyle) was mounted on a commercially available platinum-iridium stent (Cheatham Platinum, NuMed, Hopkinton, NY) (Figure 1). The stented prosthesis was then circumferentially compressed over a balloon-tipped catheter (NuMed, 25–30 mm OD, 50 mm long). The expansion of the balloon expanded the stented prosthesis to its proper shape. 

**Figure 2:**
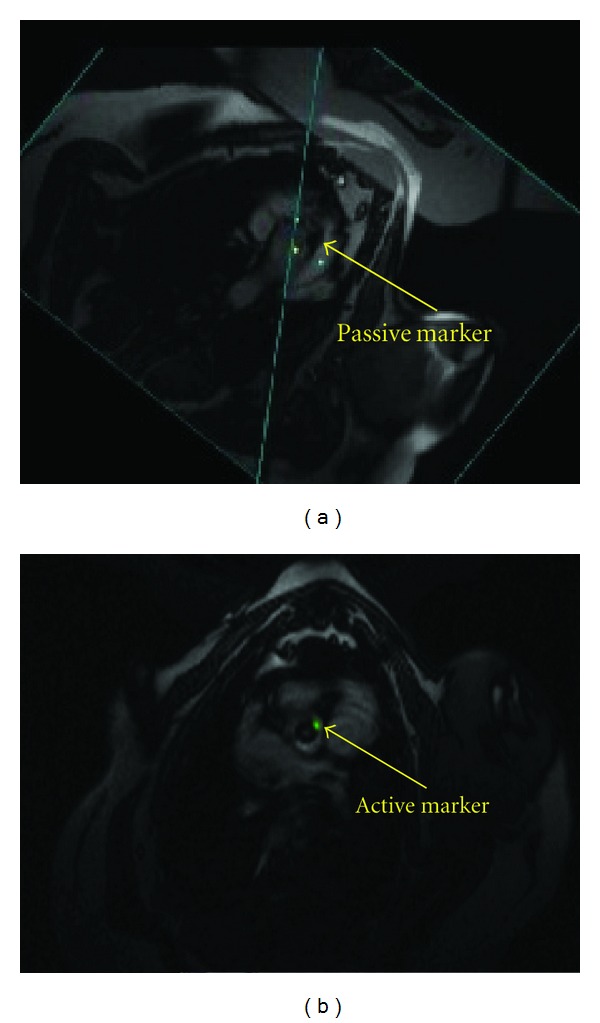
(a) Passive marker showing black signal in MRI. (b) Active marker showing bright signal and highlighted in green. These markers are used to indicate the orientation of the prosthesis in an MRI-guided aortic valve implantation procedure.

Small austenitic stainless steel fragments (0.5 mm) were welded on the side of both the balloon-expandable and self-expanding stents. This paramagnetic passive marker is visible as a dark signal in the MRI and is used to indicate the orientation of the stented prosthesis (Figure 2(a)).

A delivery device was developed for holding and delivering the stented prosthesis (Figure 1). The delivery device consists of a straight plastic rod, outside of which is a sheath protecting the stented prosthesis before it is deployed. The diameter of the delivery device is 9.5 mm and fits into a 10 mm trocar. The inner rod has a central channel for a guide wire, balloon catheter, and/or stent retrieving device. A small rubber gasket is used to prevent blood leakage from the central channel. The plastic rod can move back and forth inside the sheath. An active guide wire is embedded in a groove on the sheath. This active guide wire is shown as a bright signal in the MRI and is also used to indicate the orientation of the stented prosthesis (Figure 2(b)). There is a handle on the inner rod and the sheath, respectively, for the surgeon to hold and manipulate the delivery device. 

### 2.3. Valve Replacement Procedure

We chose Yucatan pigs (45–57 kgs) as the animal model for the preclinical studies. The principle reasons for this choice are the similarity to the cardiac anatomy of humans and suitability for long-term studies because growth is somewhat limited compared to domestic strains over the 6 months of followup.

After the large animal was intubated and anesthetized, the physician placed the trocar into the apex of the heart. Specifically using standard titanium surgical instruments via a 6-cm subxiphoid incision, the pericardium was opened and the apex of the heart was exposed. Two concentric purse strings were placed around the apex, through which a 10-mm trocar was inserted into the left ventricle. Typical time to complete this part of the procedure was 15 to 20 minutes. Standard MR sequences were performed to obtain the orientation of the heart, evaluate ventricular and valve function, and locate the native valve annulus and the origin of the coronary arteries. Prescanning also allows setting up scan planes to be used for real-time imaging during valve implantation and followup myocardial perfusion and aortic flow imaging. Three imaging planes were prescribed for real-time imaging during implantation. Two of these planes were positioned to provide long-axis views of the left ventricle, showing the right coronary artery and left main coronary artery origins, respectively. The other plane provided an axial view of the aortic valve. The coronary ostia and aortic annulus location were digitally marked. These digital marks remained visible at all times in the 3D rendering and were used for anatomic reference.

Based on the preoperative image, an appropriate sized prosthesis was selected. The prosthesis was then compressed and placed inside the outer sheath at the distal end of the delivery device. The prosthesis was aligned with the active guide wire in the sheath of the delivery device.

**Figure 3:**
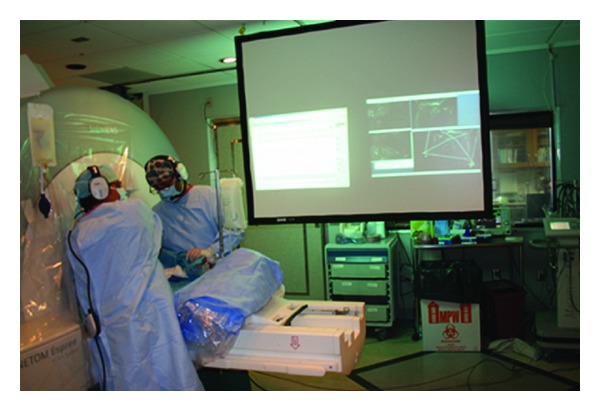
Using real-time MRI as projected onto the screen, the surgeon advances the delivery device into the LV. He can then precisely position the prosthetic valve for deployment.

The surgeon viewed the real-time imaging on a projection screen while manipulating the deployment device within the animal in the magnet (Figure 3). The prosthetic valve and delivery system were advanced through the trocar. During implantation, the axial slice was shifted as needed to visualize the device and guide proper orientation of commissures with the help of the passive and active markers. The long-axis views were interactively modified to show the path of the delivery device, while keeping the coronary origins in view. Both the active wire and the passive marker were used to identify the location and orientation of the prosthesis. The surgeon was in direct contact with the scanner operator by means of headphones and a microphone (Magnacoustics, Atlantic Beach, NY) to request changes in the imaging planes as needed.

During the procedure, the animals were monitored with an electrocardiogram, oxygen saturation, end-tidal carbon dioxide, systemic and left ventricular blood pressure, and arterial blood gas analysis.

**Figure 4:**
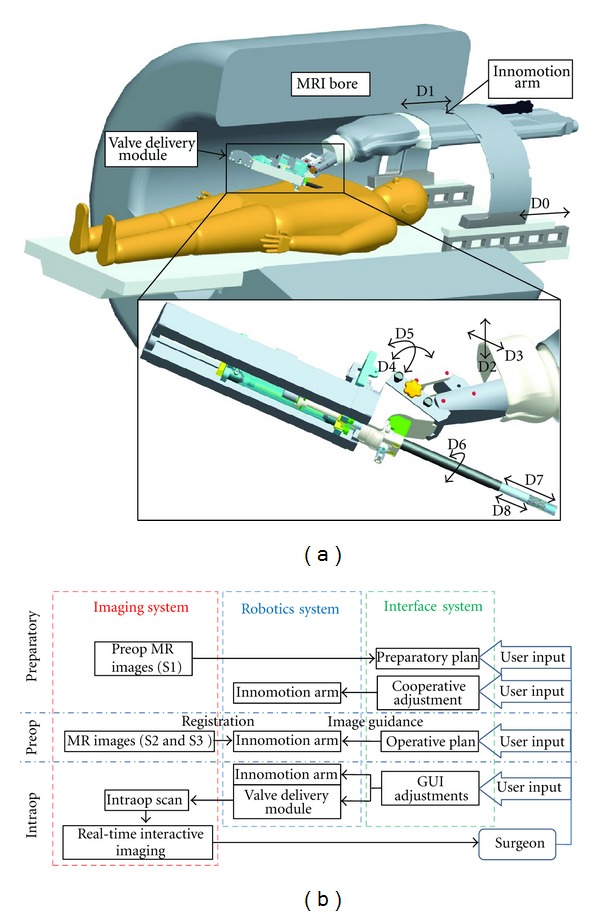
(a) A CAD sketch of the robotic system with patient inside an MRI bore. (b) Diagram showing connections between different subsystems and interactions of the physician with the system.

In a procedure using the self-expanding prosthesis, the loaded delivery device was first advanced into the ascending aorta. Upon release of the stent by retraction of the outer sheath, the chevron-like Nitinol cylinder together with bioprosthetic valve expanded to its preprogrammed diameter. Retracting and repositioning of the prosthesis were possible before the stent was fully advanced outside of the sheath (Figure 4).

In a procedure using the balloon-expandable prosthesis, the balloon is first partially inflated by using normal saline mixed 100 : 1 with an MR contrast agent Gd-DTPA (Magne vist, Berlex Inc., Montville, NJ); the position is reconfirmed to be ideal and the balloon is then fully expanded and the prosthesis deployed.

After placement of the valve, the trocar was removed and the apex closed with the purse-string sutures. After-placement images were acquired to confirm the positions of the prostheses and the valvular and heart function. Gated cine-MRI was used to assess mitral valve function and myocardial function. Phase contrast cine-MRI was used to identify flow through the new valve as well as detecting intra- or paravalvular regurgitation. An MR first-pass perfusion scan was performed during intravenous injection of Gd-DTPA contrast agent to confirm that myocardial blood flow.

### 2.4. Long-Term Evaluation

The animals were allowed to survive for long-term followup. At 1 and 3 months postoperatively, followup MRI scans and transthoracic echocardiography were acquired while at 6 months postoperatively MRI scans and confirmatory 2D and 3D transesophageal echocardiography were acquired. Retrospectively gated CINE MR, phase contrast CINE MR, and MR first-pass perfusion scanning during intravenous injection of Gd-DTPA contrast agent were repeated at those time points to confirm the position of the prostheses and the valvular and heart function. After 6 months the animals were sacrificed, and the histopathologic analyses were performed.

### 2.5. Robotic Assistance System

Based on the results seen with a surgeon and human assistant manual approach, we developed an MRI compatible robotic surgical assistant system that could more precisely deliver aortic valve prostheses [29–32]. The robotic system consists of an MRI compatible robotic arm, a valve delivery module, and user interfaces for the surgeon to plan the procedure and manipulate the robot. The CAD sketch of the 9 degree of freedom (DOF) robotic system which operates in the confined space between the MRI bore and the supine patient is shown in Figure 4. 

An MRI compatible Innomotion arm (Innomedic, Herxheim, Germany) was employed to hold the robotic module and move the valve delivery device on its planned trajectory. The robotic arm has a remote center of motion structure and its configuration fits into a standard closed MRI scanner. A robotic module was designed for manipulating a delivery device to position and deploy the prosthesis [26]. The robotic module comprises two linear joints: the translation joint and the insertion joint, as well as a rotational joint. The operations of the linear joints and the rotational joint are independent. Two linear joints can be independently or simultaneously controlled. The translation joint provides linear displacement of the delivery device along its axis. The rotation joint allows the delivery device rotating around its axis to change the orientation of the prosthesis relative to coronary ostia before it is deployed. The insertion joint moves only the inner rod of the delivery device. Sole motion of the insertion joint moves only the inner rod of the delivery device, driving the balloon-expandable prosthesis out of the protecting sheath to the desired position. Simultaneously retracting the translation joint and advancing the insertion joint at the same velocity keep the inner rod of the delivery device at its location and retracts the protecting sheath back to expose the prosthesis. This simultaneous motion will let the crimped self-expanding prosthesis expand and affix to the desired position.

To maintain image quality and prevent local heating in the proximity of the patient, the prototype module was made from nonconductive plastic materials, MR compatible pneumatic actuators (Airpel, Norwalk, CT), and magnetotranslucent fiber-optical encoders (Innomedic, Herxheim, Germany). The control PC that was placed outside of the MR room communicated with the electronic devices that control pneumatic valves and read encoder signals via the optic network.

Different interfaces—cooperative adjustment, operative plan, and interactive GUI adjustments—were implemented to suit the needs at the different phases of the procedure (Figure 4) [32]. After the physician places the trocar into the subject's heart, the Innomotion robotic arm is then mounted on the MRI table and adjusted such that its end effector is close to the trocar port. The robotic module with a fiducial rod attached is mounted on the Innomotion arm. The physician uses cooperative hands-on interface [33] to adjust the Innomotion arm to insert the fiducial rod into the trocar. Once the fiducial rod is in place, the user input sensor is detached and the robot is moved into the bore. In the preoperative phase, the patient undergoes another MRI scan for the physician to plan the trajectory of the delivery device. At the same time, another MR sequence is used for system registration. The Innomotion arm is moved to the planned trajectory, under image guidance. The fiducial rod is then replaced with the delivery device. Thus, direct access to the aortic annulus is created. In the intraoperative phase, the physician uses the visual feedback from the rtMRI and interactively adjusts and deploys the prosthesis using the robotic module via a GUI.

## 3. Results and Discussion

### 3.1. MRI Guidance

A steady-state free precession (SSFP) sequence was used with following scanning parameter: TR = 436.4 ms, TE = 1.67 ms, echo spacing = 3.2 ms, bandwidth = 1000 Hz/pixel, flip angle = 45°, slice thickness = 4.5 mm, FOV = 340 × 283 mm, and matrix = 192 × 129. The active wires were a superb indicator of the valve orientation in MRI. The passive markers on the stents also help to identify the valve orientation. These markers were somewhat difficult to visualize via MRI when the stents were fully crimped but became more apparent as the stents were deployed. Finally digital markers were placed on the images to identify landmarks and provide surgical references (e.g., light blue dots in Figure 2(a)).

**Figure 5:**
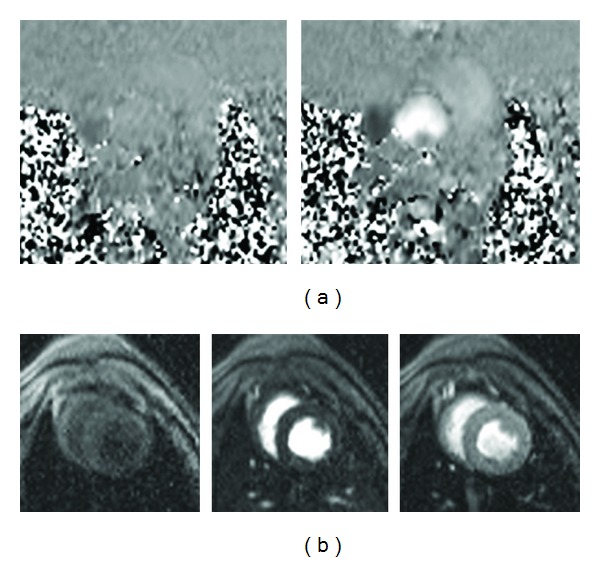
After-procedure evaluation. (a) Short-axis frames from a cine-phase contrast scan depict the blood flow through the aorta and atria after trocar removal and chest closure. These scans are used to confirm adequate valve opening and blood flow through the prosthetic valve and identify intra- or paravalvular regurgitation. Left: diastole. Right: systole. (b) A first-pass perfusion scan was performed during intravenous injection of Gd-DTPA contrast agent to confirm that myocardial blood flow was intact to all segments of the myocardium. From left to right the progression of time after venous injection of Gd-DTPA is represented.

Postplacement gated cine MRI revealed excellent myocardial function after valve implantation in both long- and short-axis views for animals in whom the valves were appropriately positioned (Figure 5). The phase-contrast CINE MR images confirmed good systolic flow with excellent valve leaflet opening and no evidence of turbulence, diastolic regurgitant flow, or paravalvular leak (Figure 5(a)). First-pass perfusion studies demonstrated adequacy of myocardial blood flow after valve placement in all animals following successful deployment. The perfusion results confirmed adequacy of blood flow at the tissue level, indicating proper valve positioning with respect to the coronary ostia (Figure 5(b)).

### 3.2. Valve Replacement

A series of short-term feasibility experiments were conducted in which 42 animals were sacrificed after valve placement and assessment by MRI. Following the acute studies, 34 animals were enrolled in chronic studies, 11 were implanted with a balloon-expandable prosthesis, and 23 were implanted with a self-expanding prosthesis. 

Total procedure time was 37 and 31 minutes for using balloon-expandable prosthesis and self-expanding prosthesis, respectively. They were not significantly different (*P *= 0.12). The time from introduction of the prosthesis into the trocar to deployment the stent is fully expanded (deployment time): 74 ± 18 seconds (mean ± std. dev.) and 60 ± 14 seconds (mean ± std. dev.), respectively. This deployment time was significantly shorter for the self-expanding prosthesis (*P* = 0.027). The procedures using balloon-expandable prosthesis take a slightly longer time because of the time used for staging the ballooninflation and the difficulty in orienting the valve knowing that once the balloon was completely inflated there was no margin to allow for adjustment.

### 3.3. Long-Term Result

**Figure 6:**
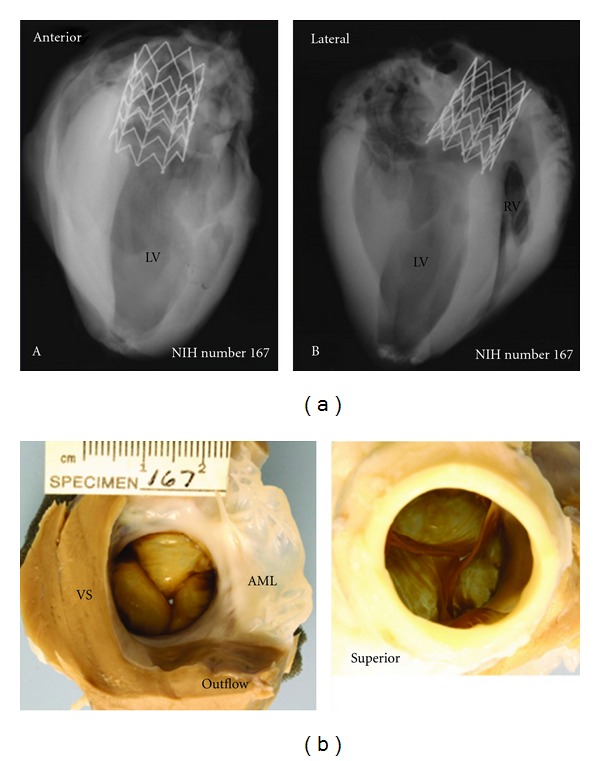
Radiographs (a) and necropsy results (b) of the hearts with the self-expanding prosthesis 6-months postimplantation. Both anterior and lateral views of the heart show an intact self-expanding stent frame without any fractures. The bottom row shows inferior and superior views of the self-expanding prosthetic aortic prosthesis; note that the entire stent frame crowns are covered as well as the annulus of the prosthetic valve is covered by opaque white tissue without pannus formation extending into the valve bases. The anterior mitral leaflet is unremarkable. The superior view of the aortic prosthetic valve shows good coaptation of the free edges of the valve leaflets. VS-ventricular septum, AML-anterior mitral leaflet.

The prostheses were successfully deployed in all of the chronic studies. Twenty-one of these survived for 6 months and were sacrificed per protocol. Postmortem pathologic analysis, after sacrifice at 6 months, verified that the implanted prostheses appeared in place in the aortic root. The prosthetic commissures were incorporated with neointimal growth continuous with the native leaflet commissures. Representative radiographs and autopsy confirmation of the self-expanding prosthesis after 6 months implantation are shown in Figures 6(a) and 6(b). 

The average strut fractures for the platinum iridium balloon-expandable stent were 5.0 ± 3.1 (mean ± std. dev.), while the average fractures for the self-expanding stent were 1.6 ± 2.5 (mean ± std. dev.) (*P *= 0.046). There was no particular pattern of strut fractures observed. The fractures are due to the stent material fatigue and the expansion, contraction, torsion between the aorta and the stent. 

### 3.4. Robotic Assistance

The MR compatibility of the entire robotic system was evaluated using a 16-cm cylindrical MR phantom inside a 1.5T Siemens Espree scanner. This imaging protocol was similar to the one we used for the cardiac intervention. The imaging series were taken with (1) phantom only and (2) robotic system placed in the magnet and running during imaging. The presence and motion of the robotic system inside the scanner were found to have no noticeable disturbance in the image. The observed SNR loss was 8.2% for the entire robotic system placed in the scanner and in motion. 

**Figure 7:**
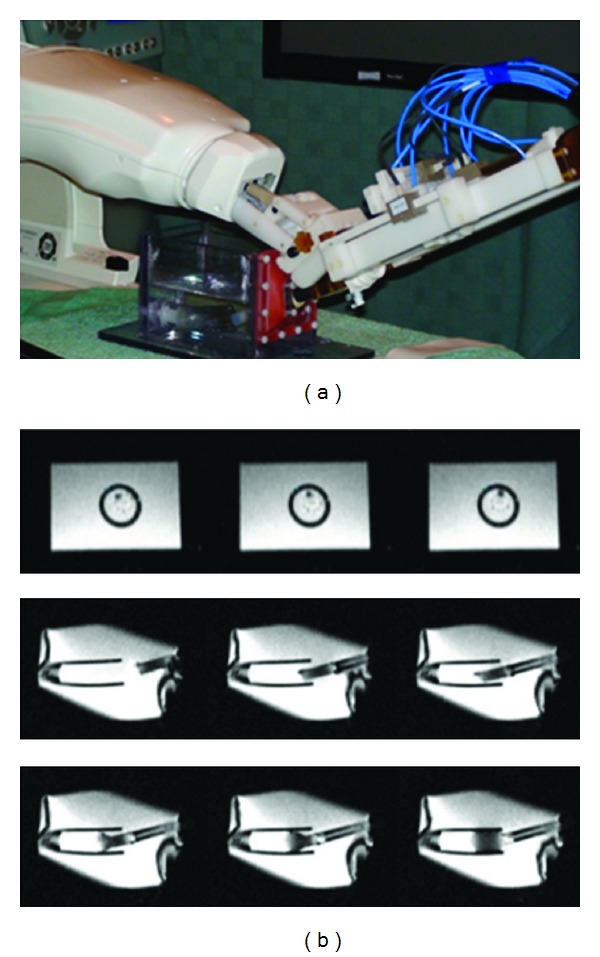
(a) Setup for system level evaluation on a phantom. The prototype of robotic valve deliver module is mounted on an Innomotion arm. (b) Sequence of MR images showing the progress of using our robotic system to place prosthesis under MRI guidance. First row shows the orientation adjustment of the prosthesis. Second row shows the position adjustment of the prosthesis. Third row shows the deployment of the self-expanding prosthesis.

We tested the robotic system on a custom-designed phantom for self-expanding prosthesis deployment (Figure 7(a)). The phantom was designed to emulate the dimensions of the valve replacement situation for testing the feasibility of the robotic system. It consisted of a plastic tube with 25-mm diameter, which served as the aorta. The diameter of the tube is the typical size of adult human aortic root. This is mounted on one side of a 200 × 100 × 100 mm water tank. A spherical joint mounted on a flexible, elastic membrane located on the opposite side of the tank served as the apex. A 12–15-mm trocar was inserted into the spherical joint. The distance from spherical joint to the end of the plastic tube was 50 mm, which is the typical distance from the heart apex to the aorta annulus as measured in the clinical scenario. The trocar insertion point had some compliance due to the mounting arrangement.

The self-expanding prosthesis requires coordinated motion between two coupled pneumatic joints, thus making it a more challenging scenario. We aimed to deploy the self-expanding prosthesis such that its proximal edge is on the edge of the tube under rtMRI guidance using robotic system.  Figure 7(b) shows the progress of the orientation adjustment and the position adjustment of the prosthesis, as well as the progress of the deployment of the prosthesis. After the prosthesis was deployed, we measured the distance between the edge of the tube and the edge of the prosthesis. The average of absolute system level error over seven trials was 1.14 ± 0.33 mm.

## 4. Discussion

Despite the requirement of a minithoracotomy, transapical aortic valve implantation is a relatively easy, safe, and straightforward technique. The short and direct access route allows excellent alignment between the prosthesis and the aortic root. With the assistance of the visualization of the active and passive markers on the devices in the MRI, the orientation and positioning of the implanted valve are more precise and predictable. 

Real-time MRI with proper parameter values provides excellent visualization for intraoperative guidance of aortic valve replacement on the beating heart. It provides better image quality and a complete view of the entire volume of interest more than other competing imaging methods, such as fluoroscopy/angiography, in which some anatomic structures are not visible, and echocardiography, in which the field of view is small and can be obscured by calcification which is frequently the source of the valvular problem. MRI-guided surgery also allows direct functional assessments to be made before, during, and immediately after valve implantation that are not obtainable by conventional imaging alone. However, the presence of a strong magnetic field of MRI scanner demands all the devices used must be MRI safe and compatible. 

Both self-expanding and balloon-expandable prostheses are used in TAVR. In our experience, self-expanding stents were easier to position and deploy thus leading to fewer complications during transapical aortic valve replacement. The intrinsic radial force of the self-expanding stent allows for even expansion of the prosthesis. As a result, the orientation of the implanted valve is more predictable. The self-expanding stent can be retrieved and repositioned before it is fully expanded; this aids precise placement and diminishes the risk and embolization. The self-expanding stent, with its specific geometric design, handles torsion better, while the balloon-expandable stent has no elasticity and the material is relatively soft leading to more frequent strut fractures.

Robot assistance can reduce the cognitive load on the physician with improved accuracy and repeatability in transapical valve replacement under MRI guidance. The high magnetic field and the confined space of an MR scanner present many technical challenges. The mechatronic components including actuators, sensors, and controllers must be able to work in an accurate, stable, and robust way in an MR environment. Materials used for a robotic system should have low magnetic susceptibilities (comparable with air, water, or human tissue), low electrical conductibility, adequate mechanical strength, and good manufacturing properties. 

The robotic system has been tested on a stable phantom. This phantom is not an ideal replica of the beating heart; but with proper anatomical dimension between the aortic annulus and the apex, it provides a reasonable situation to validate the coordinated working of the different components of the integrated system before preclinical experimentation.

The control strategy and the human machine interface for MRI compatible robot systems for medical interventions need to be studied. In the engineering of robots for medical applications, detailed analyses of the functions of the entire system, that is, robot, interfaces and application, taken as single entity, are arguably more important than the individual performance of the subsystems (robot, surgeon, interfaces, and application, separately). Thus, having a combination of more than one interface such as; an image-guided interface, console guided interface, or hands-on interface based on the specific application might yield a higher performance from the entire system.

## 5. Conclusion

Minimally invasive cardiac surgery reduces trauma and speeds recovery of the patient. It allows a cohort of patients considered to be at prohibitively high risk for undergoing standard surgical cardiac operation to potentially realize the benefits of a better functioning heart without the morbidity and mortality of a conventional operation.

However, minimally invasive cardiac surgical procedures can be technically demanding and more constrained than open procedures. Restricted vision, the complexity of instrument manipulation, and difficulty with hand-eye coordination are frequent barriers to the implementation of minimally invasive procedures. We used transapical aortic valve implantation as an example; demonstrated minimally invasive cardiac surgery can be implemented with the integration of surgical techniques, the technologies of medical images, medical devices, and robotics. The feasibility of the implantation of the transapical aortic valve under real-time interactive MRI guidance was successfully demonstrated. The long-term survival experiments further confirm that this minimally invasive surgical technique is safe and robust, ready for translation to a clinical trial.

MRI provides real-time viewing to allow guidance of procedures in the blood-filled heart without requiring cardiopulmonary bypass and cardiac arrest. Real-time noninvasive MR imaging that can provide both anatomic details and functional assessments enables the use of minimally invasive cardiac approaches that may provide patients with a less morbid and more durable solution to structural heart disease. The ability to measure cardiac function online is also an advantage to performing the minimally invasive surgery within the MR scanner. Despite its preeminent image quality, MRI has not been widely implemented in all centers. MRI equipment is expensive to purchase, maintain, and operate. A single MRI scanner can cost over 1.5 million dollars. Moreover, MRI has stringent requirements for interventional tools. Devices that are used during interventions, such as catheters, are usually not designed to be MR visible or compatible as they often contain ferromagnetic materials or long electrical conductors. 

Robotics augments the dexterity and accuracy of instrument manipulation in a confined space. The marriage of a medical imaging system and a robot makes the benefit of minimally invasive interventions substantial. An MRI compatible robotic assistant system was developed for assisting in transapical aortic valve replacement. Different interfaces were implemented to suit the needs at the different phases of TAVR procedure. The experimental results show that this robotic system can assist to smoothly deliver the prosthesis under real-time MRI guidance with high accuracy. The presence and motion of the robotic system inside the MRI scanner were found to have no noticeable disturbance to the image. The performance of using interactive interface to control the robotic system in a beating heart is under further evaluation in an animal study.

With the assistance of improvements in engineering technologies such as medical imaging, surgical navigation, and robotic devices, more cardiac surgeries can be performed in a minimally invasive fashion. We believe minimally invasive cardiac technique development is a long evolutionary process; it requires collaborative efforts of physicians and engineers to work cooperatively to fill in the technological gaps.
